# Nonlinear Absorption in 2D Ruddlesden–Popper Perovskites: Pathways to Ultrafast Optical Applications

**DOI:** 10.1021/acs.jpclett.4c01673

**Published:** 2024-09-16

**Authors:** Ja-Hon Lin, Jen-Feng Hsu, Yi-Chung Yang, ChunChe Lin, Chiung-Cheng Huang, YanQi Ge

**Affiliations:** † Department of Electro-Optical Engineering, National Taipei University of Technology, Taipei 10608, Taiwan; ‡ Department of Molecular Science and Engineering, National Taipei University of Technology, Taipei 10608, Taiwan; § Department of Chemical Engineering and Biotechnology, Tatung University, Taipei 10491, Taiwan; ∥ College of Physics and Optoelectronic Engineering International Collaborative Laboratory of 2D Materials for Optoelectronics Science and Technology, Shenzhen University, Shenzhen 518060, China

## Abstract

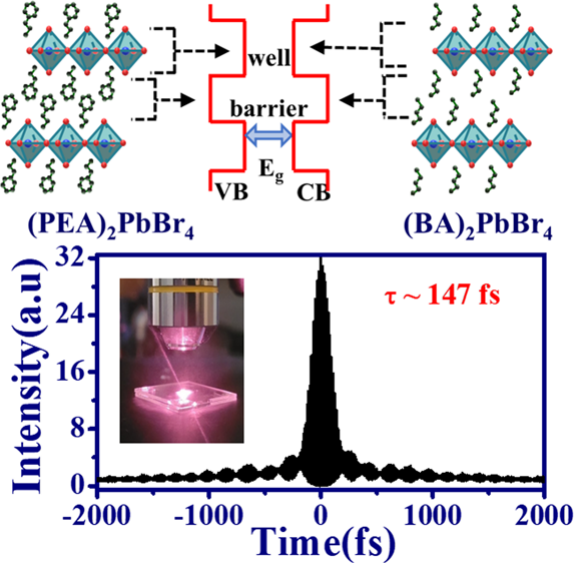

Owing to their distinctive photophysical properties resulting from their larger exciton binding energy and the influence of dielectric and quantum confinement effects, considerable research interest has been directed toward two-dimensional Ruddlesden–Popper halide perovskites (2D RP-HPs). Particularly, 2D RP-HPs exhibit exceptional multiphoton absorption (MPA) effects that reveal versatile applications. In this work, two-photon absorption (2PA) and three-photon absorption (3PA) in 2D RP-HPs, specifically (PEA)_2_PbBr_4_ and (BA)_2_PbBr_4_ platelets, have been demonstrated through their photoluminescence spectra under multiphoton excitation, revealing a power law relationship with excitation energy. On the other hand, polarization-dependent MPA measurements are also conducted to obtain their anisotropy properties. The excellent 2PA and 3PA effects of 2D RP-HPs have found applications in ultrafast optics to construct fringe-resolved autocorrelation traces, enabling the retrieval of pulse duration not only passively mode-locked Ti:sapphire at 800 nm but also Yb-doped fiber lasers at 1030 nm.

Due to their superior characteristics, such as a high photoluminescence quantum yield (PLQY), low-cost solution-processable halide perovskites (HPs) have become one of the most attractive materials. However, the poor stability remains a crucial challenge that requires immediate improvement for the commercialization of perovskites in the near future. Numerous strategies, such as ligand modification,^1^ embedding techniques such as defect passivation,^2,3^ and phase stabilization,^4^ have been proposed to prevent their degradation in environmental conditions. On the other hand, two-dimensional Ruddlesden–Popper halide perovskites (2D RP-HPs) provide an alternative solution, which possess a layered stacking structure composed of alternative inorganic components and bulky or long-chain monovalent organic cations, such as C_3_H_7_NH_3_^+^ (PA, propylammonium), C_4_H_9_NH_3_^+^ (BA, butylammonium), and C_6_H_5_C_2_H_4_NH_3_^+^ (PEA, phenethylammonium).^5^ In comparison with three-dimensional HPs, the presence of organic cations in 2D layered HPs can reduce the possibility of photodegradation and oxidation reactions, thereby enhancing environmental stability.^6^ Additionally, 2D-layered HPs exhibit remarkable features such as larger exciton binding energy,^7^ higher nonlinear optical properties,^8^ and wavelength tuning possibility by adjusting the number of perovskite layers.^9^ Due to their exceptional property, 2D RP-HPs have been utilized in applications such as photodetector,^10^ solar cell,^11^ and light emission diode (LED).^12^

Multiphoton absorption (MPA)^13^ is one of the interesting and useful nonlinear optical phenomena resulting from the interaction between light and material. In this process, carriers are excited to higher energy levels by means of the simultaneous absorption of multiple photons.^14^ Theoretically, MPA arises from odd order nonlinear polarization , which can be expressed as  = , where ϵ_0_ is the vacuum permittivity; χ^(*m*)^ denotes the *m*^*th*^-order (*m* > 1 and *m* is an odd integer) optical susceptibility, and *E⃗* is the electric field of the incident light. For instance, two- and three-photon absorption (i.e., 2PA and 3PA) are associated with the imaginary component of the third- and fifth-order nonlinear optical susceptibility (i.e., χ^(3)^ and χ^(5)^), respectively.^15^ Contrary to linear absorption, which employs continuous-wave (CW) lasers as a light source, MPA is induced by low-power, high-intensity infrared ultrashort pulsed lasers. This unique approach offers several advantages, including deeper penetration depth,^16^ higher axial resolution,^17^ and a lower damage threshold.^18^ Consequently, MPA finds applications in various fields, such as microscopy techniques,^19^ photodynamic therapy, microfabrication of 3D materials,^20^ and biomedical imaging.^21^

The Z-scan method, first proposed in 1991, has been widely adopted due to its simple structure as a technique for determining third-order nonlinear parameters, such as the nonlinear refractive index and the 2PA coefficient.^22^ Utilizing this technique, larger 2PA coefficients have been observed from both organic–inorganic hybrid perovskite single crystals^23^ and films^24^ and all-inorganic perovskites like CsPbBr_3_ QDs.^25^ This enables the utilization of 3D HPs in photodetectors,^26,27^ upconversion lasers,^28^ optical power limiters,^29,30^ and ultrafast optics.^25,31^ In contrast to 3D HPs, the unique multiquantum-well structure of 2D RP-HPs, characterized by an alternating arrangement of organic and inorganic components, induces quantum and dielectric confinement effects, thereby endowing them with superior nonlinear optical properties. For instance, Liu et al.^8^ illustrated a notably large 2PA coefficient about 211.5 cm/MW from 0.95 μm thick phenylethylamine lead iodide (PEA)_2_PbI_4_, which is at least 1 order of magnitude larger than those of 3D perovskite films. Zhou et al.^32^ demonstrated remarkable 2PA and 3PA from 2D layered perovskite, namely, (C_4_H_9_NH_3_)_2_(CH_3_NH_3_)Pb_2_I_7_ (BA_2_MAPb_2_I_7_), which makes it suitable for photodetection in the near-infrared spectrum range.

On the other hand, the material with intrinsic anisotropic property can be used to develop polarization-sensitive optoelectronic devices and optical components within the fields of optoelectronic devices,^33^ the laser industry,^34^ and display technology.^35,36^ Through the pump polarization-dependent two-photon excitation PL measurement, the anisotropic property from 3D organic–inorganic perovskite single crystal such as MAPbBr_3_, MAPbI_3_,^23^ and all-inorganic perovskite CsPbBr_3_^37^ were investigated. Additionally, the anisotropic nonlinear optical properties of 2D layered RP perovskites have been elucidated, encompassing single-layer materials,^38^ such as (C_6_H_5_(CH_2_)_2_NH_3_)_2_PbI_4_ (PEPI), (C_6_H_11_NH_3_)_2_PbI_4_ (C6H11), and (C_4_H_9_NH_3_)_2_PbI_4_ (C4PI), as well as multilayer material like BA_2_MAPb_2_I_7_.^32^ In 2019, Dhanabalan et al.^39^ proposed a straightforward method for growing 2D layered perovskite platelets using aliphatic or aromatic amines as an inorganic layer serving as a spacer between organic layers constructed by corner-sharing  octehedra. They demonstrated that 2D RP-HPs platelets derived from aliphatic amines such as (PEA)_2_PbBr_4_, and platelets derived from aromatic amines such as (BA)_2_PbBr_4_, exhibited higher photoluminescence quantum yields (PLQYs) than other organic moieties.^39^ However, reports on the nonlinear optical properties of 2D layered perovskites, particularly their applications in ultrafast optics, remain scarce. In this study, the MPA effect of anisotropic 2D RP-HPs such as (PEA)_2_PbBr_4_ and (BA)_2_PbBr_4_ was investigated for the first time, to the best of our knowledge, using a femtosecond Ti:sapphire laser and a Yb-doped fiber laser as light sources. Through 2PA and 3PA PL, we not only retrieve the anisotropic parameters of the 2D RP-HPs but also explore their applications in retrieving the pulse duration of ultrashort pulsed lasers through autocorrelation techniques.

Two-dimensional perovskites, with a unified formula RAm_2_MA_*n*__– 1_Pb_*n*_Br_3__*n*+ 1_, is shown in Figure 1(a), where RAm is the general ammonium ion resulting from the protonation of the selected primary amine, such as PEA and BA, MA corresponds to methylammonium ions, and *n* is the number of layers in the structures. In this study, the synthesized material reveals monostacked layers (*n* = 1), as neither MA source nor other ions were added during the synthesis process.^39^ In order to analyze the morphology of the sample surface, the crystalline state of (PEA)_2_PbBr_4_ and (BA)_2_PbBr_4_ platelets are shown from the images of the scanning electron microscope (SEM, scale bars:100 μm) in Figures 1(b) and 1(c), respectively. To confirm their crystallinity, we show the X-ray diffraction (XRD) patterns in Figure 1(d). Both structures show the well-defined diffraction peaks with equidistant angular spacing from the diffraction of plane (00*h*, *h* = 2, 4, 6, ...). This suggests that the 2D layered RP-HPs are formed by the alternating arrangement of organic and inorganic layers along the *c*-axis direction, showcasing a relatively high crystalline quality and orientation. Due to the periodic distribution of diffraction peaks, we can calculate the spacing of the inorganic layers according to the Bragg formula: *nλ* = 2*d* sin θ, where *n* is the diffraction order, *d* is the spacing of the inorganic layers, and θ is the angle of incidence. When considering the first-order diffraction peak with θ = 5.32° in (PEA)_2_PbBr_4_ and θ = 6.32° in (BA)_2_PbBr_4_ platelets, the estimated period of each organic–inorganic layer is about 1.66 and 1.4 nm, respectively, which closely matches previously reported results of about 1.51 nm^40^ and 1.4 nm.^41^ By the thermogravimetric analysis (TGA), both 2D layered materials reveal a feature of a three-step degradation process, and the thermal stability was up to 240 and 220 °C for the (PEA)_2_PbBr_4_ and (BA)_2_PbBr_4_, respectively (Table S1 in Supporting Information).

**Figure 1:**
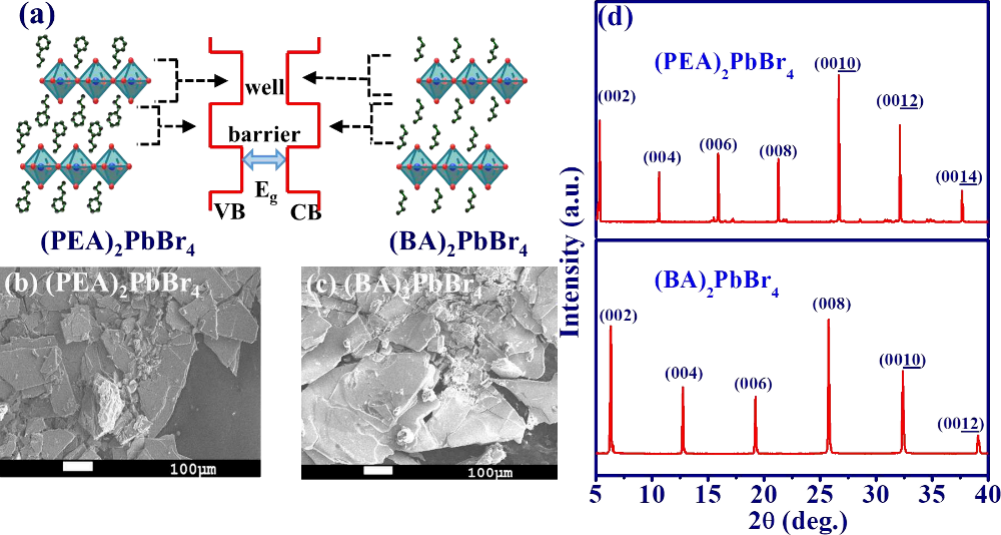
(a) Crystal structures of 2D-layered RP perovskites such as (PEA)_2_PbBr_4_ (Left hand side) and (BA)_2_PbBr_4_ (right-hand side) with their electronic band structure (middle), and SEM images (Scale bars: 100 mm) of (b) (PEA)_2_PbBr_4_ and (c) (BA)_2_PbBr_4_, as well as (d) X-ray diffraction (XRD) for the (PEA)_2_PbBr_4_ (top) and (BA)_2_PbBr_4_ (bottom).

The reflectance (red solid curve) and one-photon absorption photoluminescence (1PA PL, blue solid curve) spectra, excited by the 325 nm CW He–Cd laser, of 2D layered perovskite platelets are shown in Figure 2(a). Similar to the previous results,^39^ it is clear to see the two dips, i.e., *D*_1_ and *D*_2_ (Table S2 in Supporting Information), which can be attributed to the interior and surface absorption.^42^ Through Gaussian decomposition, three emission peaks, namely, *P*_1_, *P*_2_, and *P*_3_, are obtained, as shown in Figure S2 (Supporting Information). Here, *P*_1_ and *P*_2_ are attributed to interior and surface emissions, while *P*_3_ results from the defect state, as depicted in Table S2 (Supporting Information). Besides, the photon energy of emission peaks under one photon excitation for both 2D-layered RP-HPs are larger than the band-edge (Figure S3 in Supporting Information), termed the above bandgap emission.^23^ The relatively large binding energy of approximately 110.3 and 80.1 meV for (PEA)_2_PbBr_4_ and (BA)_2_PbBr_4_, respectively, can be determined from temperature-dependent PL measurement (Figures S4 and S5).

**Figure 2:**
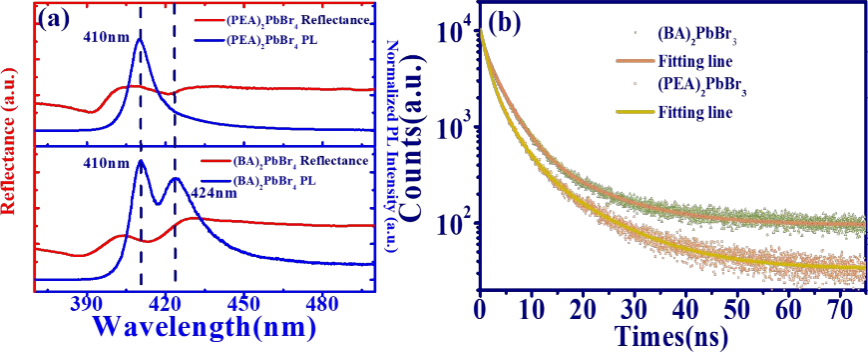
(a) Reflectance (red line) and 1PA PL spectrum of (PEA)_2_PbBr_4_ and (BA)_2_PbBr_4_ flake (Blue line). (b) 1PA photon decay trace of (PEA)_2_PbBr_4_ and (BA)_2_PbBr_4_ platelet.

In Figure 2(b), the photon decay traces of (PEA)_2_PbBr_4_ (orange squares) and (BA)_2_PbBr_4_ (green squares) platelets are derived using the time-correlated single photon counting (TCSPC) technique employing a picosecond pulsed laser (pulse duration τ = 50 ps, λ_*p*_ = 372 nm, PicoQuant Inc.) as the excitation source. Through the fitting of the triexponential function,^43^ the fast decay constants τ_1_ and τ_2_ are due to nonradiative recombination such as Auger recombination, while the slow decay constant τ_3_ is attributed to the single-exciton recombination,^44^ as listed in Table 1. Considering scaling factor *A*_1_, *A*_2_, and *A*_3_, the estimated average decay time constants τ_*ave*_ of (PEA)_2_PbBr_4_ about 4.98 ns and (BA)_2_PbBr_4_ about 5.82 ns are comparable to previous findings,^45,46^ respectively.

**Table 1:** Summary of reported photon lifetime from different types of (PEA)_2_PbBr_4_ and (BA)_2_PbBr_4_ in previous literature

Sample	τ_1_(ns)	*A*_1_(%)	τ_2_(ns)	*A*_2_ (%)	τ_3_(ns)	*A*_3_(%)	τ_*a*_*vg* (ns)
(PEA)_2_PbBr_4_^45^	1.23	—	5.31	—	—	—	—
(BA)_2_PbBr_4_^46^	1.45	72	5.64	34	33.37	0.9	6.82
(PEA)_2_PbBr_4_(Our work)	1.14	60.6	3.40	33.56	11.59	5.83	4.98
(BA)_2_PbBr_4_(Our work)	0.87	57.91	3.46	34.51	11.83	7.56	5.82

In the following, we investigated MPA PL of 2D layered perovskite. Theoretically, 2PA and 3PA result from the simultaneous absorption of two or three photons, facilitating the transition of electron from valence band to the conduction band, as illustrated in Figures 3(a) and 3(d), respectively. In the following, 2PA and 3PA PL of 2D-layered perovskite platelets were investigated using a passively mode-locked (PML) Ti:sapphire laser and a Yb-doped fiber laser (YDFL) with peak wavelength at 800 and 1030 nm as an excited light source. The corresponding photographs are also shown on the right side of Figures 3(a) and 3(d). As the excited intensity increases, the evolution of 2PA PL spectra of (PEA)_2_PbBr_4_ and (BA)_2_PbBr_4_ platelets are shown in Figures 3(b) and 3(c). Compared to the 1PA PL emission in Figure 2(a), the highest emission peaks (*P*_*p*_) of 2PA PL from 2D-layered perovskites are shifted to the longer wavelength, specifically, *P*_*p*_ = 427 nm in (PEA)_2_PbBr_4_ and 424 nm in (BA)_2_PbBr_4_. The red-shift is attributed to the deeper penetration depth for the longer excitation wavelength to result in the enhancement of self-absorption effect.^47^ The plot of integrated PL as a function of exciting intensity is shown in the inset of Figures 3(b) and 3(c). By the fitting of the formula: *I*_*MPA*_ = *αI*_*exc*_^*n*^, where α is a constant, and exponent *n* is 2.10 and 2.06 for (PEA)_2_PbBr_4_ and (BA)_2_PbBr_4_ platelet, respectively. It indicates that the peak intensity *I*_*2PA*_ reveals quadratic relation to the excitation intensity *I*_*exc*_, illustrating that 2PA dominates under the excitation of a fs Ti:sapphire laser.

**Figure 3:**
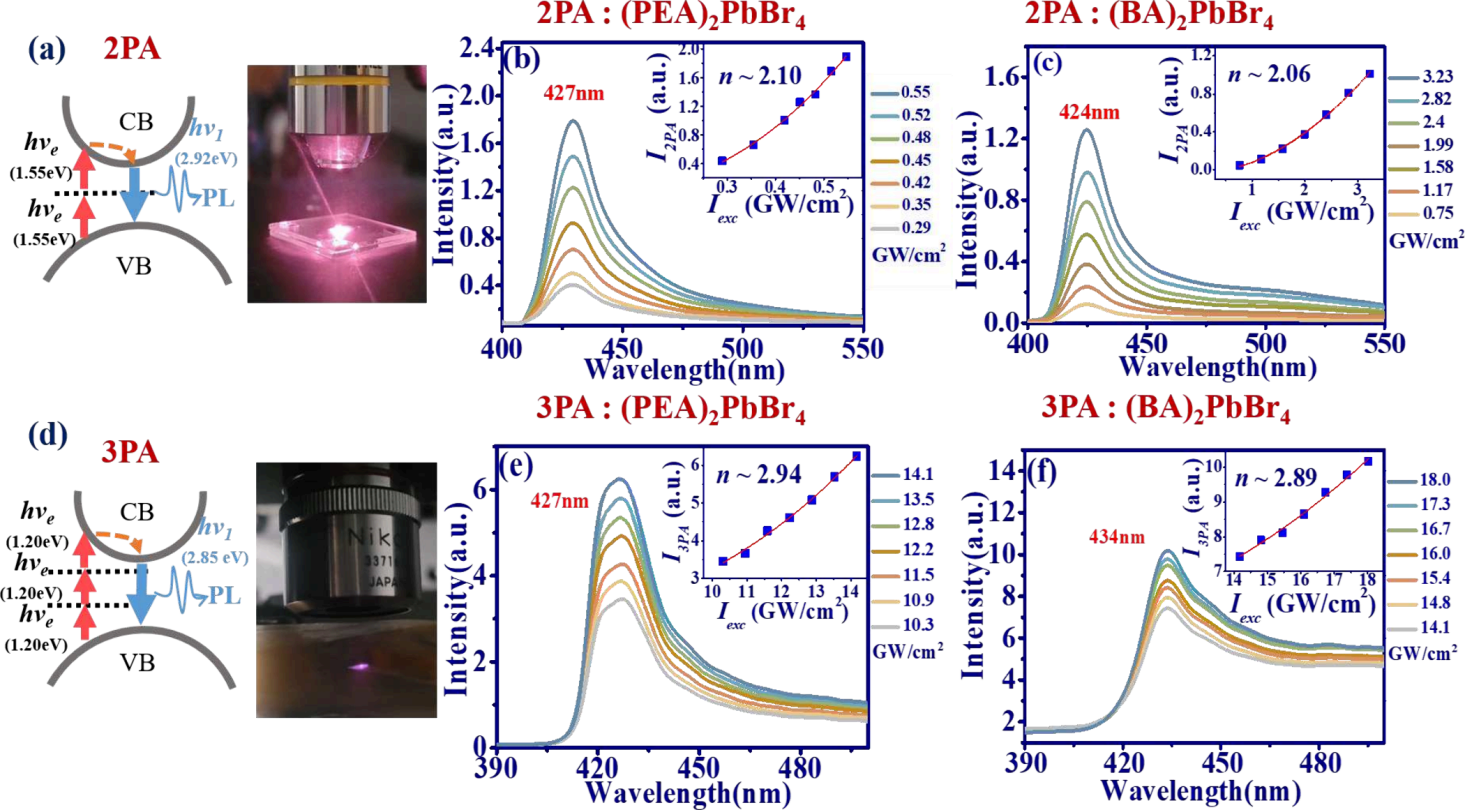
MPA PL of 2D RP-HPs. (a) Schematic plot to illustrate the energy diagram of (left) and the corresponding photo of sample under excitation (right) for 2PA. Evolution of 2PA PL spectrum as pump intensity increases for (b) (PEA)_2_PbBr_4_ and (c) (BA)_2_PbBr_4_ platelets (the inset plot shows the PL peak intensity (*I*_*2PA*_) versus excitation intensity (*I*_*exc*_)). (d) Schematic plot to illustrate the energy diagram of (left) and the corresponding photo of sample under excitation (right) for 3PA. Evolution of 3PA PL spectrum as pump intensity increases for (e) (PEA)_2_PbBr_4_ and (f) (BA)_2_PbBr_4_ flakes (the inset plot shows the PL peak intensity (*I*_*3PA*_) versus excitation intensity (*I*_*exc*_)).

In addition, the 2PA coefficient β was obtained through open aperture Z-scan measurements (Figure S6 in Supporting Information), yielding approximately 94.61 cm/GW for (PEA)_2_PbBr_4_ and 268.1 cm/GW for (BA)_2_PbBr_4_. These values are larger than those obtained from 3D perovskites, such as films and single crystals (Table S4 in the Supporting Information). Two-photon absorption, originating from the third-order nonlinear polarization of electrons, can be enhanced by a factor related to the enhancement of the oscillator strength in the quantum well structure of 2D RP-HPs. The enhancement factor of third-order susceptibility can be expressed with the relation^8,48^
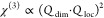
1where *Q*_dim_ and *Q*_loc_ represent the enhancements in oscillator strength resulting from dimensional confinement and exciton localization, respectively. Owing to the specific quantum and dielectric constraints, the carriers can undergo substantial confinement within the ultrathin atomic inorganic layer for 2D RP-HPs. As a result, a strong light and matter interaction, such as exciton–photon coupling^49^ and oscillator strength of exciton transition,^50^ can be achieved to induce a nonlinear absorption effect.

Similarly, the evolution of 3PA PL as the excitation intensity increases is shown in Figures 3(e) and 3(f). The emission peak of (PEA)_2_PbBr_4_ remains at 427 nm (Figure 3(e)), but the emission peak of (BA)_2_PbBr_4_ exhibits a red-shift from 424 to 434 nm in Figure 3(f). This shift is owing to the even deeper penetration depth using 1030 nm YDFL as a light source. Through the fitting of emission peak intensity *I*_*3PA*_ as a function of excited intensity (inset of Figures 3(e) and 3(f)), the obtained exponent *n* around 3 accounted for the effect of 3PA. The photostability of 2D RP-HPs has been investigated under 1PA, 2PA, and 3PA conditions. Due to the deeper penetration depth associated with longer excitation wavelengths, which leads to improved thermal diffusion, the material under MPA shows better photostability than under 1PA (refer to Figure S7 in the Supporting Information).

The anisotropic properties are intrinsic characteristics of halide perovskites, revealing the potential for specific applications. However, the anisotropy of 2D layered perovskites under 2PA and 3PA has rarely been discussed. In the following section, we explore the anisotropy of 2D RP-HPs using pump polarization-dependent MPA PL spectroscopy.^32^ Here, the pump pulse propagates along the [001] direction of the 2D layered perovskites. When the pump polarization was rotated within the in-plane of the platelets, the peak wavelength of the MPA PL spectra remained unchanged, while the peak intensity varied (refer to Figures S8 and S9 in the Supporting Information). The polar plots of angle-dependent intensity variation from 2PA and 3PA–PL spectra exhibit similar four-petal patterns in Figures 4(a) and (b). They can be fitted well using^23^
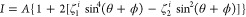
2Here, the constant *A* is proportional to the high-order susceptibility, the coefficients ζ_1_^*i*^ and ζ_2_^*i*^ represent the anisotropy coefficients of MPA (*i* = 2 and 3 refers to the 2PA and 3PA processes, respectively), θ is the angle between the pump polarization vector and the crystallographic axis [100], and ϕ is the angular offset.^38^ It is important to note that the anisotropy coefficients ζ_1_^*i*^ and ζ_2_^*i*^ approach each other as the crystal structure transitions to the cubic case. In the case of a material being completely isotropic, ζ_1_^*i*^ and ζ_2_^*i*^ become zero.^23^ Through the accurate fitting of eq 2, depicted by the tangerine and teal solid lines in Figures 4(a) and (b), the anisotropy coefficients of both 2D layered materials under both 2PA and 3PA conditions can be derived, as summarized in Table 2. A distinct anisotropy is evident in both (PEA)_2_PbBr_4_ and (BA)_2_PbBr_4_, exhibiting triclinic (*P*1) structure^51^ and orthorhombic (*Pbca*) structure at room temperature.^52^

**Figure 4:**
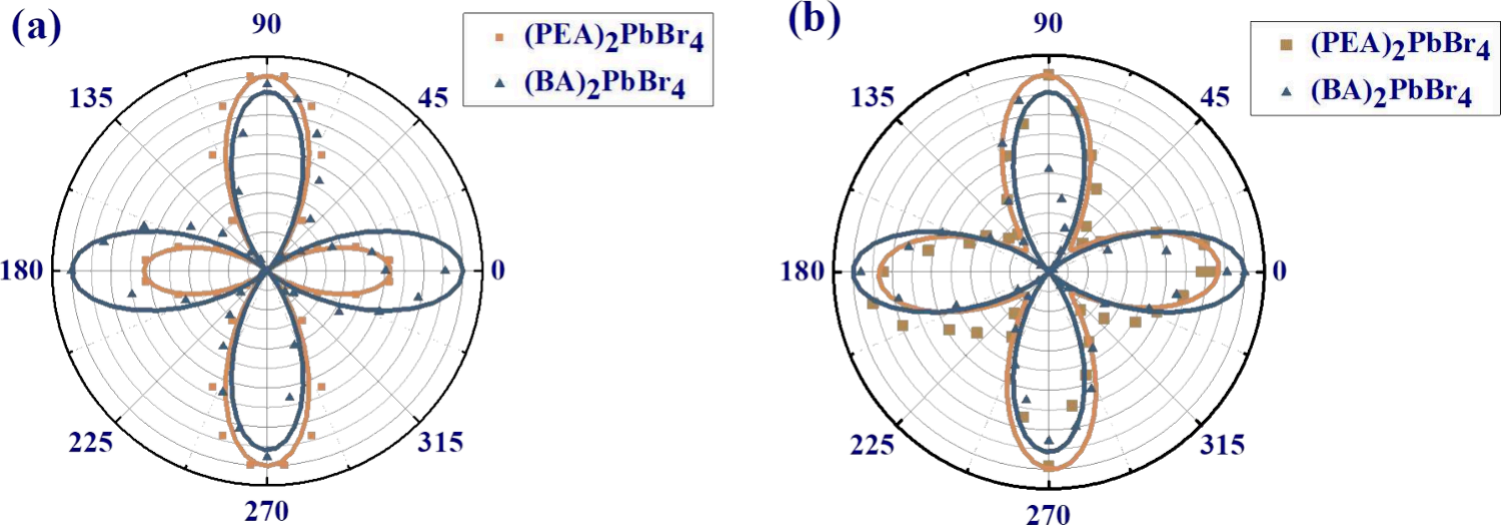
Polarization-dependent NLO measurements for (PEA)_2_PbBr_4_ (tangerine square line) and (BA)_2_PbBr_4_ (indigo blue triangle line) platelets. Polar plot of (a) 2PA PL signal excited via fs Ti:sapphire laser and (b) 3PA PL signal excited via fs Yb-doped fiber laser.

**Table 2:** Summary of the reported anisotropy coefficients ζ_1_ and ζ_2_ from perovskites

Sample	ζ_1_^2^	ζ_2_^2^	ζ_1_^3^	ζ_2_^3^
MAPbI_3_^23^	0.17	0.26	-	-
MAPbBr_3_^23^	0.72	0.72	-	-
PEPI^38^	1.10	1.12	1.09	1.25
C6H11^38^	1.39	1.50	0.94	1.01
C4PI^38^	0.92	1.10	1.41	1.58
BA_2_MAPb_2_I_7_^32^	1.18	1.01	-	-
(PEA)_2_PbBr_4_(our work)	0.61	0.68	1.79	1.71
(BA)_2_PbBr_4_(our work)	0.57	0.55	1.33	1.30

Due to their excellent MPA effect, organic–inorganic and all-inorganic perovskite materials have been widely applied in various purposes such as optical limiting,^53^ optical switching,^54^ and ultrafast optics.^30^ In contrast to second-harmonic crystals, MPA does not require phase matching, making it easy to set up and provide a response across a wide range of wavelengths for ultrafast measurement. To measure the pulse duration of an ultrafast laser, an autocorrelator comprising an interferometer and a nonlinear medium is commonly used to obtain the fringe-resolved autocorrelation trace (FRAC). In this setup, the incident pulse is split into two replicas by a beam splitter (BS) and then recombined within a nonlinear optical medium, as shown in Figure 5(a). The induced intensity of MPA PL from a nonlinear medium is related to nonlinear absorption processes such as 2PA or 3PA. As a result, the signal detected by the PMT is proportional to the sum of pulse intensities, i.e., the overlap of the two pulses, in the nonlinear medium. By movement of one of the mirrors (M2), the phase delay between the two pulses can be adjusted to produce the interference fringe, known as self-autocorrelation.

**Figure 5:**
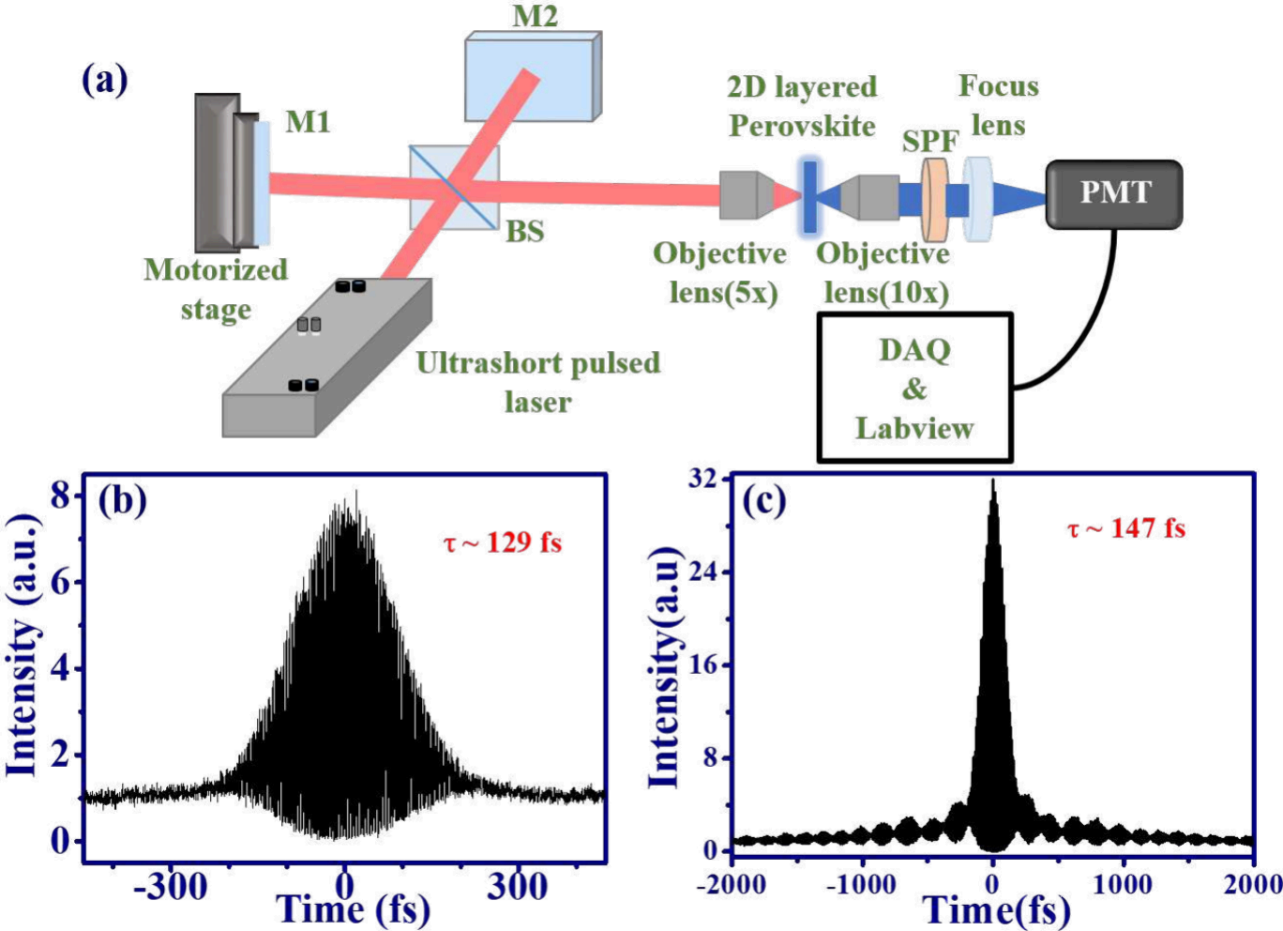
Application of 2D RP-HPs on ultrafast optics. (a) Setup of interferometric autocorrelator, (b) FRAC trace of fs Ti: sapphire laser based on 2PA of (PEA)_2_PbBr_4_ platelets, and (c) FRAC trace of fs Yb-doped laser based on 3PA of (BA)_2_PbBr_4_ platelets.

Various optoelectronic devices, such as LED^55^ and GaAsP photodiode,^56^ have been used as a 2PA medium for the pulse duration measurement of ultrashort pulsed laser. On the other hand, the FRAC of PML Ti:sapphire laser has been obtained by means of 2PA PL from ZnO nanorods,^57^ MAPbBr_3_ single crystals,^31^ and CsPbBr_3_ QDs.^25^ In comparison to the techniques relying on 2PA, FRAC based on 3PA is more sensitive and offers the ability to measure asymmetric pulse shapes without the direction-of-time ambiguity.^58,59^ However, there are relatively few reports related to the 3PA FRAC measurement. To validate the application of MPA in 2D-layered HPs for ultrafast optics, the FRAC traces of both a PML Ti:sapphire and a PML Yb-doped laser were measured by the autocorrelator as shown in Figure 5(a). Figures 5(b) and 5(c) exhibit the obtained FRAC based on 2PA PL of (PEA)_2_PbBr_4_ and 3PA PL of (BA)_2_PbBr_4_ under excitation power of 60 and 150 mW, respectively. Consistent with the theoretical prediction,^60^ the peak-to-background ratios for the 2PA and 3PA FRAC traces were approximately 8:1 and 32:1, respectively. Thus, we obtain the pulse duration of Ti:sapphire laser and Yb-doped laser are approximately 129 fs (fwhm ∼244.7 fs) and 147 fs (fwhm ∼198.7 fs), respectively.^61^

In summary, the MPA effect of 2D layered Ruddlesden–Popper halide perovskite (RP-HPs) platelets has been demonstrated under excitation by both a PML Ti:sapphire laser and a Yb-doped fiber laser, with peak wavelengths of 800 and 1030 nm, respectively. In contrast to the PL emission observed under 1PA, the emission peaks under 2PA and 3PA for 2D-layered perovskites exhibit a noticeable red shift due to the self-absorption effect from the deeper penetration depth of longer excitation wavelengths. Additionally, the output intensities of 2PA and 3PA PL increase quadratically and cubically with the pump intensity, respectively. We also demonstrate the anisotropic characteristics in both (PEA)_2_PbBr_4_ and (BA)_2_PbBr_4_ at room temperature through the polarization-dependent MPA PL measurements. Based on their superior 2PA and 3PA properties, 2D layered perovskite platelets have been chosen as outstanding nonlinear media for constructing interferometric autocorrelators in ultrafast optical applications. In addition to retrieving the pulse duration of a passively mode-locked Ti:sapphire laser, approximately 129 fs based on 2PA, a fringe-resolved autocorrelation trace based on 3PA, with a peak-to-background ratio of around 32:1, was obtained to characterize a 147 fs Yb-doped fiber laser.

## Experimental Section

### Chemicals and Reagents

Lead(II) oxide (PbO, 99.9%) and phenylethylamine (PEA, 99%) were purchased from Acros Organics. Butylamine (BA, 99.5%) was purchased from Sigma-Aldrich. Hydrobromic acid (HBr, Alfa Aesar, 47–49%), hypophoaphoeous acid (H_3_PO_2_, Alfa Aesar, 50%), and Lead(II) bromide (PbBr_2_, Alfa Aesar, 98+%) were purchased from Alfa Aesar. All of the chemicals were used without purification.

### Synthesis of (PEA)_**2**_PbBr_**4**_ Flake

One mmol portion of PbO and 0.25 mL of PEA were added in a polytetrafluoroethylene (PTFE) container with 5.0 mL of HBr and 5.0 mL of H_3_PO_2_. The container was placed in a muffle furnace and kept at 160 °C for 30 min. Crystals were obtained by cooling the solution to room temperature naturally. The (PEA)_2_PbBr_4_ crystals can be separated through filtration.

### Synthesize of (BA)_2_PbBr_4_ Flake

Starting solutions were prepared by combining a stoichiometric amount of 95 mg of PbBr_2_ in a vial with 60 μL of HBr and 1 mL of acetone. The resulting mixture was vigorously stirred to obtain a clear and transparent solution. Subsequently, 0.1 mL of butylamine was added to the solution. After a few minutes, crystals began to grow. These crystals were collected and left to dry overnight on filter paper. All experiments were carried out at ambient room temperature.^39^

### One Photon Absorption (1PA) PL Measurement

A CW He–Cd laser (IK3301R-G, KIMMON KOHA Inc.) with a central wavelength of 325 nm was used as a light source. The excited source was reflected by an edge filter (SP01-355RU-25, Semrock Inc.) and focused onto the sample by a UV objective lens (20X, LMU-20X-UVB, Thorlabs Inc.) with a focal length of 4.1 mm. The 1PA PL emission from the sample was collected by the object lens and passed through the edge filter. Finally, the PL emission was dispersed by a monochromator (iHR 320, Horiba Inc.) and detected by a photomultiplier (R928, Hamamatsu Inc.).

### Multiphoton Absorption (MPA) PL Measurements

For the 2PA PL measurement, the excitation source is a linearly polarized PML Ti:sapphire laser (Tsunami 3941-X1BB, Spectra-Physics Inc.) with a central wavelength of 800 nm. The incident pulsed light inside the microscope was reflected by an edge filter (790 nm short-pass, FF01–790/SP-25, Semrock Inc.). An objective lens (10×, Olympus Inc.) was utilized to focus the excitation source onto the sample and capture the 2PA PL emission generated from the sample. Subsequently, the emission light passed through the edge filter once again and was guided by a fiber into a monochromator (iHR 320, Horiba Inc.), where it was detected by an electrically cooled charge-coupled device (CCD) (Syncerity, Horiba Inc.). For the polarization-dependent 2PA PL measurement, a half-wave plate was employed to adjust the orientation of the pump polarization. A similar experimental setup was employed for the 3PA PL measurement, but a PML Yb-doped laser (KASMORO-1030, mRadian Femto Sources Co., Ltd.) operating at 1030 nm was used as the light source. Moreover, the edge filter was replaced with a ZT720spxxr (Chroma Inc.).

### Autocorrelator

The autocorrelator consisted of a beam splitter (2PA: FABS-800–45P-PW-1006-UV, CVI Laser Optics Inc. and 3PA: UFBS50502 from Thorlabs Inc.) and two reflective mirrors (M1 and M2). The time delay between two pulses was produced through a linear motorized stage with a maximum displacement of approximately 12 mm, which corresponds to a time delay of 80 ps. The pump pulse was focused onto the 2D layered RP perovskite by utilizing an objective lens with a magnification factor of 5x. After the residual pump pulse was blocked through a short-pass filter, the MPA PL emitted by the sample was gathered using a 10x objective lens and subsequently detected by a photomultiplier tube (PMT, Hamamatsu Inc.) through a lens. The electrical signal generated by the PMT was further processed by using a data acquisition (DAQ) card (National instrument Inc.).

## Supporting Information Available

TGA curve, 1PA PL, Tauc plot, Temperature-dependent PL, open-aperture Z-scan trace, Polarization dependent 2PL, Polarization dependent 3PL (PDF)
