# Supplementary material for: Nonlinear Absorption in 2D Ruddlesden–Popper Perovskites: Pathways to Ultrafast Optical Applications
Source: J Phys Chem Lett. 2024 Sep 16;15(38):9644–51. doi: 10.1021/acs.jpclett.4c01673 (PMC11440594; doi:10.1021/acs.jpclett.4c01673)
Supplement: Supplementary file 1 — jz4c01673_si_001.pdf [file jz4c01673_si_001.pdf]

Supporting Information

Nonlinear Absorption in 2D Ruddlesden–Popper Perovskites: Pathways to Ultrafast Optical Applications

Ja-Hon Lin [*,†] *Jen-Feng Hsu,*[†] *Yi-Chung Yang,* [†] *ChunChe Lin*, [*,‡]
Chiung-Cheng Huang ,[¶] *YanQi Ge*[*,§]

[†]Department of Electro-Optical Engineering, National Taipei University of Technology, Taipei 10608, Taiwan
[‡] Department of Molecular Science and Engineering, National Taipei University of Technology, Taipei 10608, Taiwan
[¶]Department of Chemical Engineering and Biotechnology, Tatung University, Taipei 104, Taiwan
§ College of Physics and Optoelectronic Engineering &International Collaborative Laboratory of 2D Materials for Optoelectronics Science and Technology, Shenzhen University, Shenzhen 518060, Chin
E-mail: jhlin@ntut.edu.tw; clin0530@mail.ntut.edu.tw; geyanqi@szu.edu.cn

Thermogravimetric analysis (TGA)

The thermal stability and heat resistance of 2D RP-HPs were investigated through thermogravimetric analysis (TGA) under a nitrogen atmosphere. The samples were heated from room temperature to 800°C at a ramp rate of 10°C/min. The weight loss (red curve) and derivative weight (blue curve) of $(PEA)_2PbBr_4$ and $(BA)_2PbBr_4$ platelets with increasing temperature are illustrated in Figures S1 (a) and (b). These figures showcase a three-step degradation process and demonstrate thermal stability up to 240°C and 220°C, respectively, as summarized in Table S1.

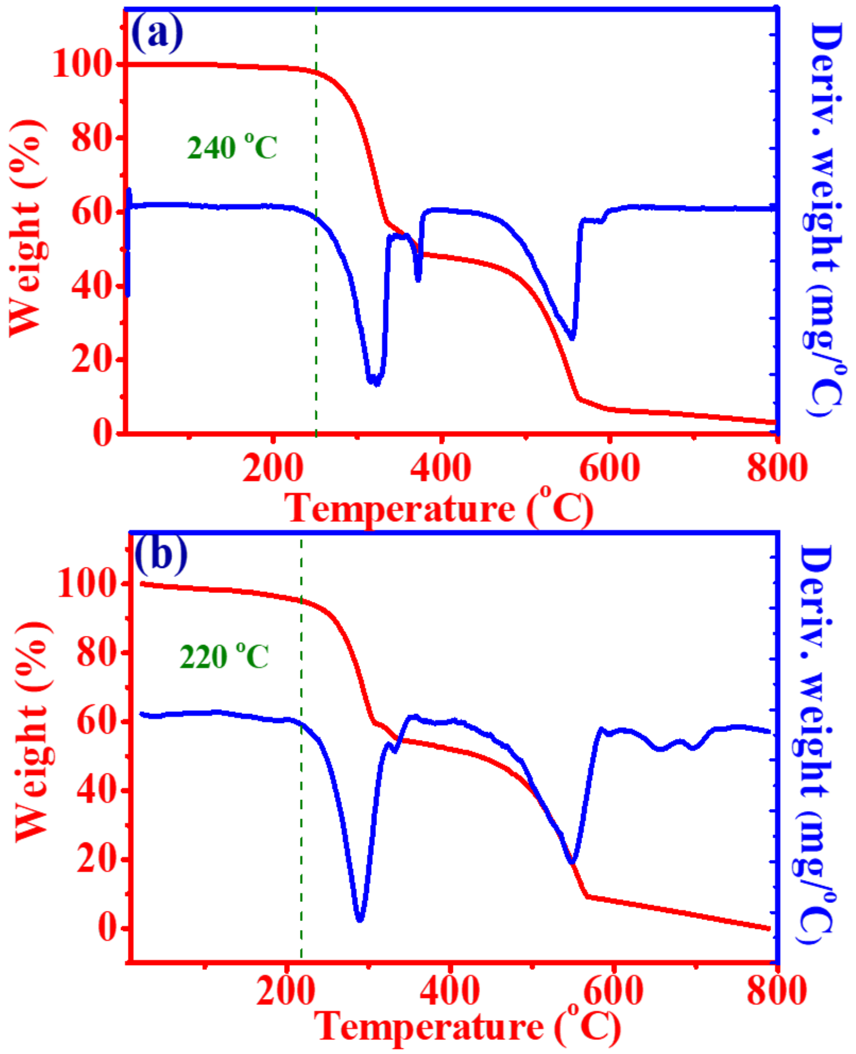

Figure S1. TGA curve and corresponding first derivative for the (a) $(PEA)_2PbBr_4$ and (b) $(BA)_2PbBr_4$ platelets.

Table S1. The weight loss and derive weight of 2D $(PEA)_2PbBr_4$ and $(BA)_2PbBr_4$ platelets as temperature increase and thermal stability characteristic.

Sample	thermal stability(˚C)	WL_1	WL_2	WL_3
**$(PEA)_2PbBr_4$**	240	42.2%	8.2%	42%
**$(BA)_2PbBr_4$**	220	35.39%	4.09%	46.29%

Reflectance and the decomposition of 1PA PL spectrum

The reflectance spectrum shown in Figs. 2(a) and 2(b) (red solid line) display two dips at D_1 = 391 nm /3.16 eV (or 387.7 nm/ 3.19 eV) and D_2 = 422 nm/2.93 eV (or 413.2 nm/ 3.00 eV) of $(PEA)_2PbBr_4$ and $(BA)_2PbBr_4$, respectively. These dips are due to the interior and surface absorption of 2D layered RP perovskite platelets. Through Gaussian decomposition, the OPA PL spectra of 2D layered RP-HPs exhibit three emission peaks, denoted as P_1, P_2, and P_3, as listed in Table 1

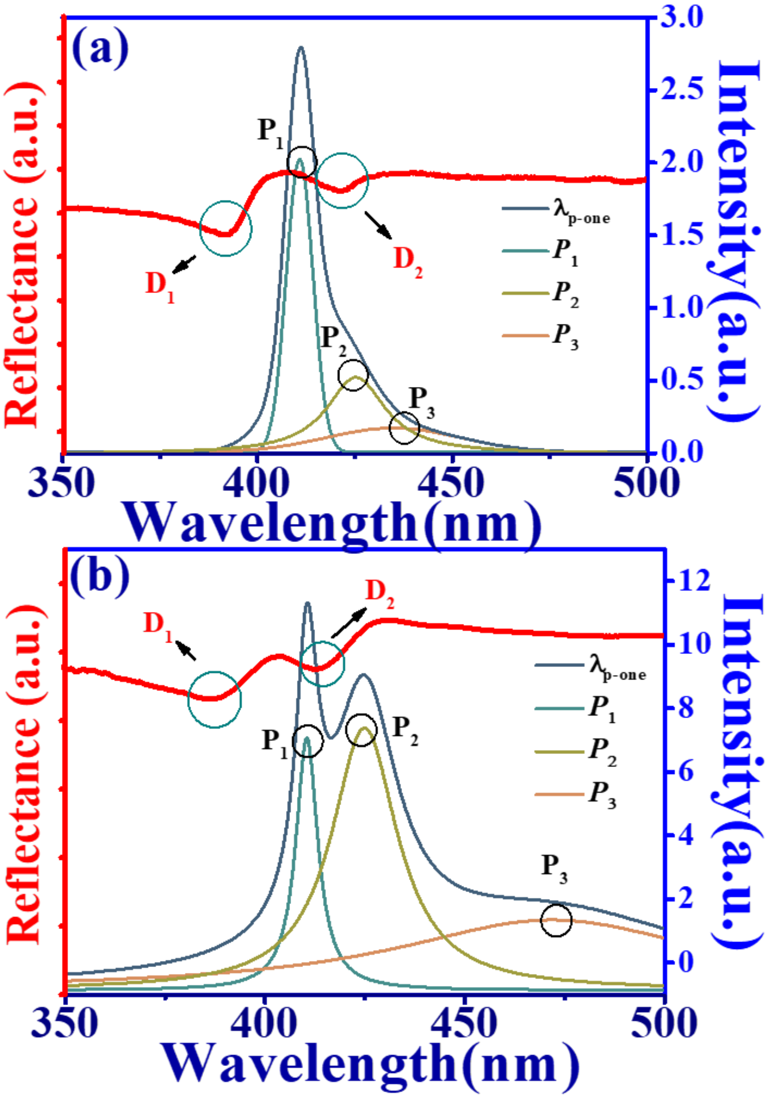

Figure S2. 1PA PL spectrum of (a) $(PEA)_2PbBr_4$ and (b) $(BA)_2PbBr_4$ platelets for the decomposition using three Gaussian functions.

Table S2. OPA PL spectrum of $(PEA)_2PbBr_4$ and $(BA)_2PbBr_4$, the decomposition using three Gaussian functions

Sample	P_1(nm/eV)	P_2(nm/eV)	P_3(nm/eV)	D_1(nm/eV)	D_2(nm/eV)
**$(PEA)_2PbBr_4$ (our work)**	410.8/3.01	427.0/2.90	434.7/2.85	391.59/3.16	422.0/2.93
**$(BA)_2PbBr_4$ (our work)**	410.4/3.02	424.1/2.92	472.4/2.62	387.7/3.19	413.2/3.00

1PA PL spectrum and Tauc plot

The emission peaks of the OPA PL spectra (blue line) are 3.022 eV (410.32 nm) for $(PEA)_2PbBr_4$ in Fig. S3(a)) and 3.021 eV (410.46 nm) in Fig. S3(b) for $(BA)_2PbBr_4$, respectively. The bandgap energy (E_g) of 2D RP-HPs can be determined using the Tauc formula: $(\alpha h\nu)^2 = A_0(h\nu - E_g)$ where $h\nu$ is the photon energy, A_0 is the slope of the curve of the Tauc plot, α is the absorption coefficient, and E_g is the bandgap of the sample. From the tauc plot in Figs. S3(a) and S3(b) (red solid line), the band gaps of $(PEA)_2PbBr_4$ and $(BA)_2PbBr_4$ are approximately 2.83 eV (438 nm) and 2.83 eV (438 nm).

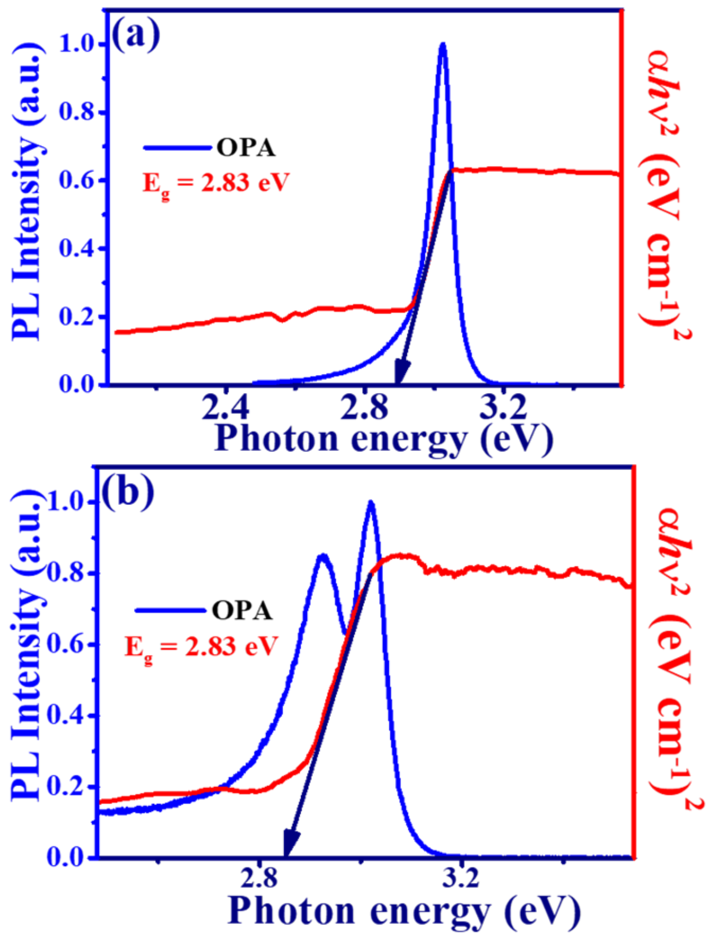

Figure S3 1PA PL (blue line) spectrum and Tauc Plot (blue line) to obtain band gap of (a) $(PEA)_2PbBr_4$ and (b) $(BA)_2PbBr_4$ flake.

Temperature dependent PL spectrum

The temperature-dependent PL was measured using a CW He-Cd laser (IK3301R-G, KIMMON KOHA Inc.) with a central wavelength of 325 nm as a light source. The sample was mounted in a cryogenic system (ST-500-UC, JANIS Inc.) to achieve precise temperature control using a temperature controller (Model 335, Lake Shore Inc.). A lens with a focal length of 3 cm was employed to focus the excitation light onto the sample.

- **Evolution of spectrum (un-normalization) as temperature increases**

Pseudo color map of temperature dependent PL spectra of $(PEA)_2PbBr_4$ is shown in Fig. S4(a). Consistent with previous findings [1], the variation of PL intensity of $(PEA)_2PbBr_4$ can be divided into three regimes, namely R-I (78 K to 100 K), R-II (100 K to 150 K), and R-III (150 K to 300 K). Figure S4(b) displays the PL spectrum of $(PEA)_2PbBr_4$ at distinct temperatures ranging from 78 K to 300 K.

- In R-I (78 K ~ 100 K), the spectrum exhibits a red shift of the emission peak and a slight decrease in peak intensity as the temperature rises.
- In R-II (100 K ~ 150 K), the emission peak shows a 9 meV red shift from 3.044 eV to 3.035 eV. The emission peak intensity increases with temperature due to the thermal activation of carriers [1].
- As the temperature increases above 150 K (150 K ~ 300 K), the peak wavelength continuously red shifts from 3.035 eV to 3.022 eV, while the peak intensity significantly decreases with temperature.

Figure S4 (c) shows the integrated intensity as a function of reciprocal of temperature (1/*T*, *T*=150 to 300 K). The integrated PL intensity as a function of temperature can be ascribed using the Arrhenius equation [S-1]:

$$I(T) = \frac{I_0}{1 + Ae^{\frac{-E_b}{kT}}}, \qquad (S-1)$$

where I_0 is the PL intensity at 0 K, E_b is the binding energy, k is the Boltzmann constant and A is constant. Based the well-fitting of Eq. (S-1) (red line in Fig. S4(b)), the binding energy E_b of $(PEA)_2PbBr_4$ is approximately 110.3 meV.

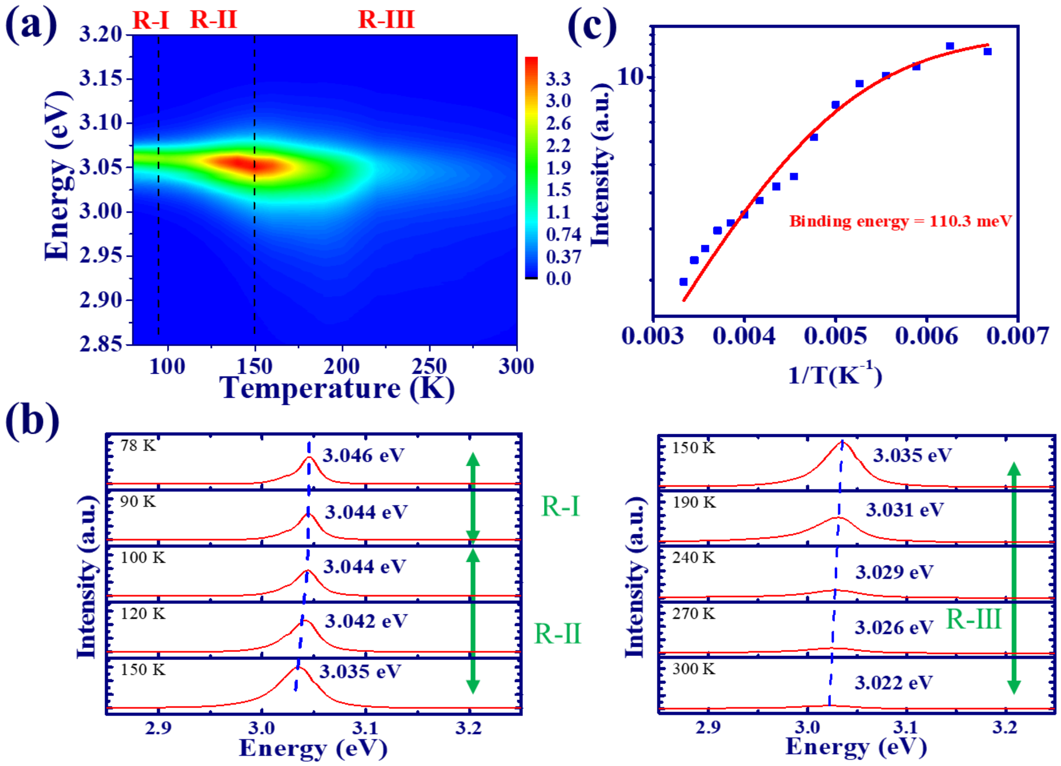

Figure S4. Temperature-dependent PL measurement of $(PEA)_2PbBr_4$ platelets. (a) Pseudo color map of temperature dependent PL spectra, (b) evolution of PL spectra as a function of temperature, (c) integrated intensity as a function of reciprocal of temperature (1/T).

Pseudo color map of temperature dependent PL spectra of $(BA)_2PbBr_4$ platelets as a function of temperature (ranging from 78 K to 300 K) is presented in Fig. S4 (a). It is clear to see the enhancement of PL intensity with increasing temperature, which is similar to the previous result [2]. Figure S4(b) show the variation of PL spectrum at different operation temperature, which indicating the emission peak of $(BA)_2PbBr_4$ was fixed at 3.024 eV. Figure S4 (c) shows the integrated intensity as a function of reciprocal of temperature (1/T, T=78 to 300 K). Based on the well-fitting of Eq. (S-1) (red line in Fig. S5(b)), the binding energy Eb of $(BA)_2PbBr_4$ is about 80.1 meV.

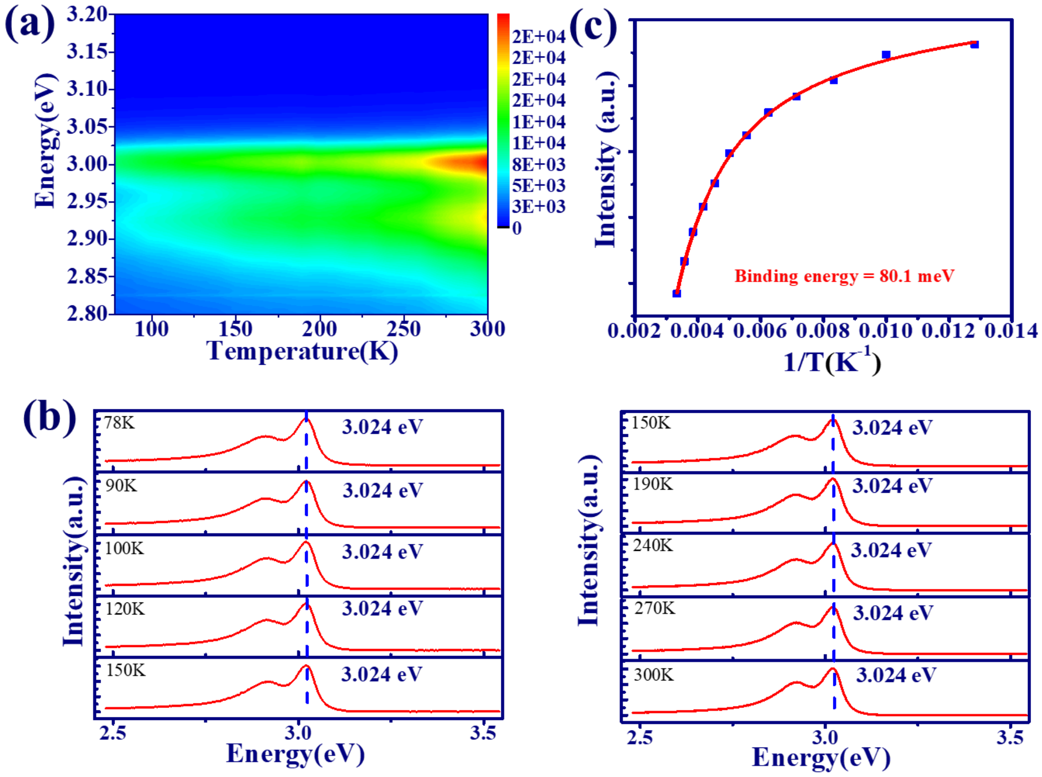

Figure S5. Temperature-dependent PL measurement of $(BA)_2PbBr_4$ platelets. (a) Pseudo color map of temperature dependent PL spectra, (b) evolution of PL spectra as a function of temperature, (c) integrated intensity as a function of reciprocal of temperature (1/T).

Two-photon absorption coefficient from Z-scan measurement

Z-scan measurement is the most widely used method to obtain 2PA coefficients owing to its simple experimental setup and ease of data interpretation. In this study, a linearly polarized PML Ti:sapphire laser with a central wavelength of 800 nm was employed as the light source, operating at a repetition rate of 80 MHz. The incident pulsed light was divided into two parts by a polarization beam splitter. The reflected light was detected by a photo-diode as a reference signal, and the transmitted light was focused onto the sample through a 12.5 mm focal length objective lens (5x, Nikon Inc.). The sample was moved back and forth along the z-axis by a motorized translation stage. A long-pass filter (665 nm long-pass, FGL665, Thorlabs Inc.) behind the sample was used to block the PL from the sample. The transmitted light was detected by the other photo-diode. Two lock-in amplifiers in combination of optical chopper were used to increase the signal to noise ratio from two photo-diodes.

Figure S4 shows the inverse transmittance ($1/T_0$) of open-aperture Z-scan trace as a function of pump intensity. Theoretically, the normalized transmission T_0 can be expressed by

$$T_0(I) = \frac{e^{-\alpha L}}{\beta I L_{eff} + 1}, \qquad (S-2)$$

where I is the peak intensity of pulsed light onto the sample, L is the thickness of sample, $\alpha(cm^{-1})$ is the absorption coefficient (at 800 nm), β is the two-photon absorption coefficient, and L_{eff} is the effective length of sample given by

$$L_{eff} = \frac{1 - e^{-\alpha L}}{\alpha}. \qquad (S-3)$$

Here, the thickness L of $(PEA)_2PbBr_4$ and $(BA)_2PbBr_4$ platelets about 0.11mm and 0.21mm, respectively. By the fitting of Eq. S-4, the nonlinear absorption coefficient β of the 2D $(PEA)_2PbBr_4$ at 800 nm is about 94.61 cm/GW and 268.1 cm/GW.

The inset figure of Figs. S6(a) and (b) show the symmetric normalized transmittance of 2D $(PEA)_2PbBr_4$ and $(BA)_2PbBr_4$ platelets from open aperture Z-scan measurement with pump intensity I of 0.41 GW/cm^2 and 0.19 GW/cm^2, respectively. Table S3 listed the reported 2PA coefficient from different kind of perovskite including 3D and 2D materials.

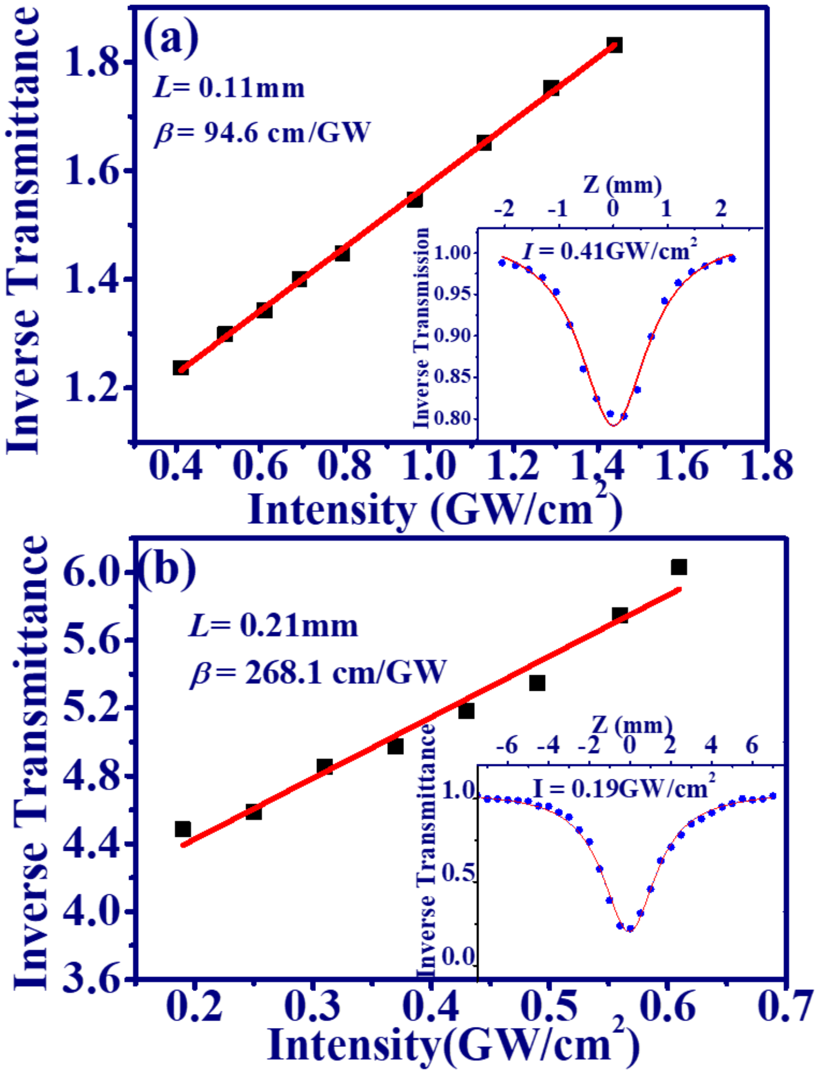

Figure S6. Inverse transmission (black squares) versus pump intensity from (a) $(PEA)_2PbBr_4$ and (b) $(BA)_2PbBr_4$ (red lines: fitted curve base on Eq. S-2). The inset figure shows the open-aperture Z-scan trace (red line: theoretical fitted curve).

Table S3: Summary of reported 2PA coefficients from 2D and 3D perovskite

**Perovskite**	**β (cm/MW)**	**λ_{exc} (nm)/Rep. rate (Hz)**	**Ref**
**Iodide perovskite**			
$(PEA)_2PbI_4$ flake (0.95 μm)	211.5	800 / 1k	[3]
$(PEA)_2PbI_4$ film (1.94 μm)	12.6	800 / 1k	[3]
$MAPbI_3$ SC (190 mm)	2.3×10^{-2}	1064 / 50	[4]
$CsPbI_3$ nanocrystals (2.4 nm)	2.73×10^{-4}	800 / 1k	[5]
**Bromide perovskite**			
$MAPbBr_3$ film (~200 nm)	5×10^{-2}	800 / 1k	[6]
$MAPbBr_3$ SC (1 mm)	8.6×10^{-3}	800 / 76M	[7]
$CsPbBr_3$ SC (1 mm)	5×10^{-3}	1000 / 50	[8]
$(PEA)_2PbBr_4$ flake (0.11 mm)	9.46×10^{-2}	800 / 80M	our work
$(BA)_2PbBr_4$ flake(0.21 mm)	2.68×10^{-1}	800 / 80M	our work

literature

The photostability of 2D layered Perovskite

The photostability of $(PEA)_2PbBr_4$ and $(BA)_2PbBr_4$ under 1PA, 2PA and 3PA are investigated using CW He-Cd laser, fs Ti:sapphire laser, and fs Yb-doped fiber laser as light source. Figures S7(a)-(c) display the long-term intensity decay monitoring of $(PEA)_2PbBr_4$ (red circles) and $(BA)_2PbBr_4$ (black squares) under 1PA, 2PA and 3PA. After one-hour of continuous pulse excitation, the produced 2D layered perovsktie reveal less intensity decay under multiphoton excitation compared to 1PA. This is due to the deeper penetration depth of the 2D layered perovskite with longer excitation wavelengths, which enhances thermal diffusion and improves photonic stability.

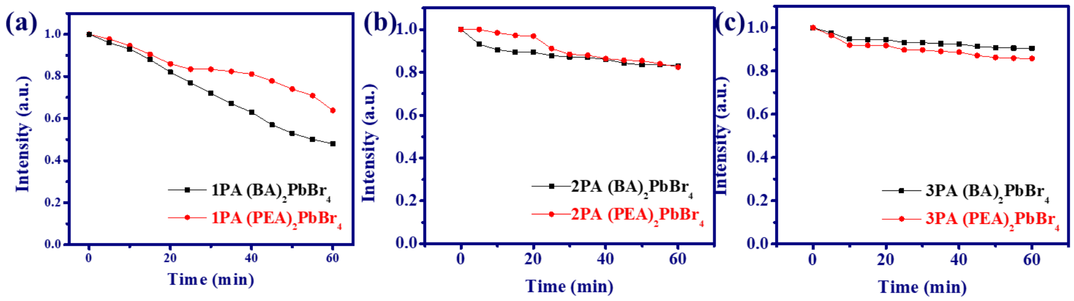

Figure S7 The recorded intensity decay of $(PEA)_2PbBr_4$ and $(BA)_2PbBr_4$ under (a) 1PA (a) 2PA and (b) 3PA.

The polarization dependence of the MPA PL spectra of the 2D layered perovskite

The pump polarization dependent 2PA and 3PA PL spectrum of $(PEA)_2PbBr_4$ and $(BA)_2PbBr_4$ by using an fs Ti:sapphire laser and an Yb-doped fiber lasers as a light sources are shown in Figs. S8 and S9.

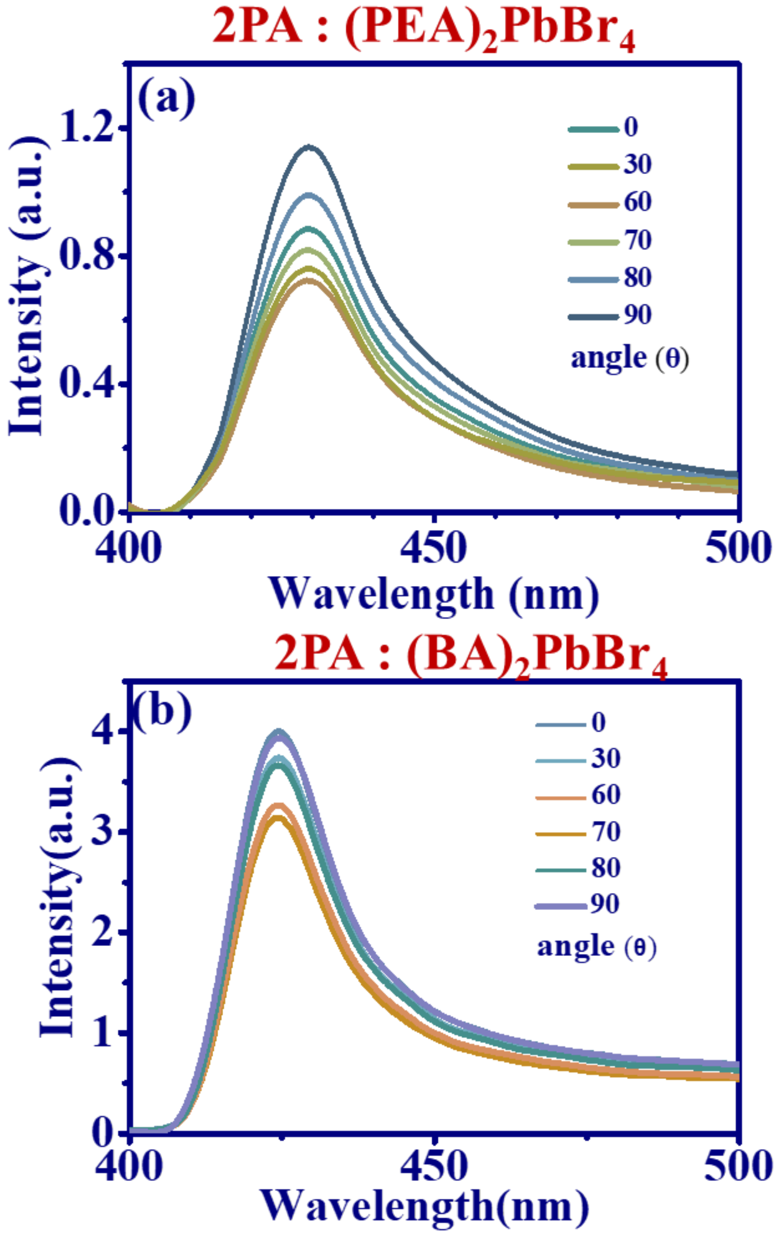

Figure S8. The polarization dependence of the 2PA PL spectra of the (a) $(PEA)_2PbBr_4$ and (b) $(BA)_2PbBr_4$ platelets was recorded at rotation angles (θ) from 0° to 90°

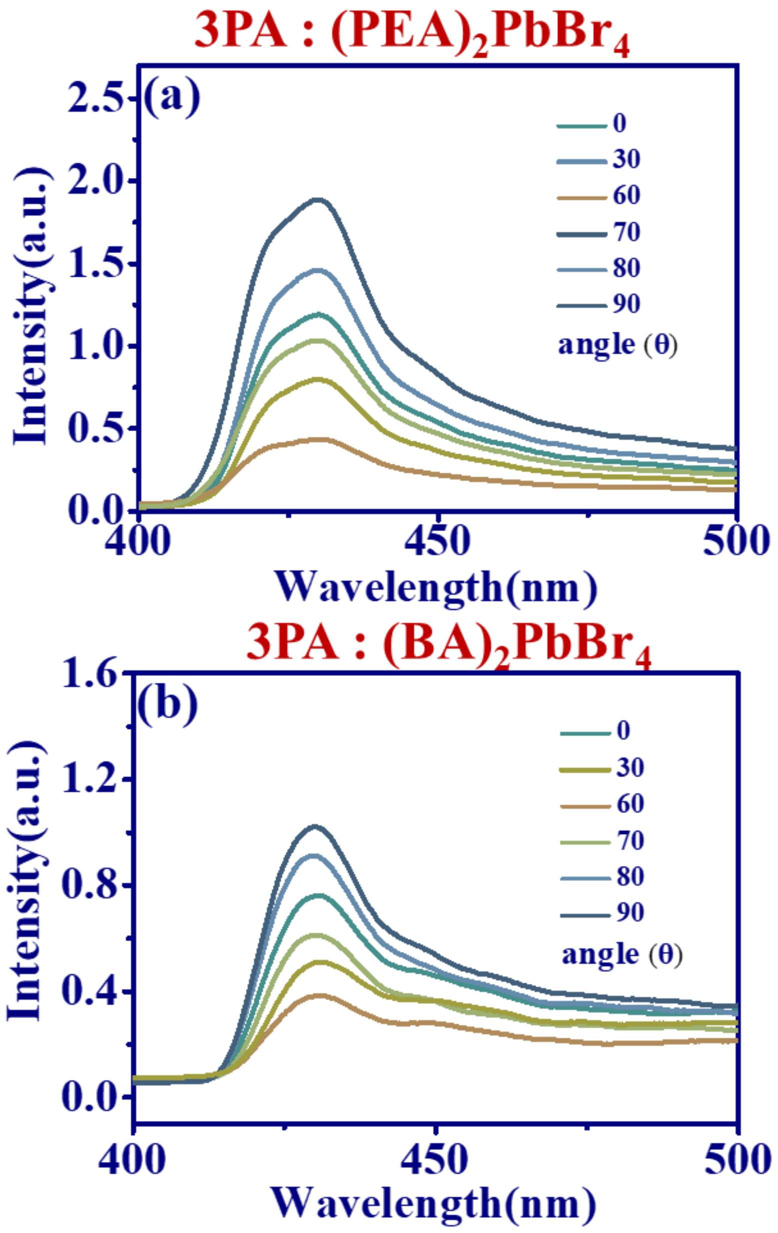

Figure S9. The polarization dependence of the 3PA PL spectra of the (a) $(PEA)_2PbBr_4$ and (b) $(BA)_2PbBr_4$ platelets was recorded at rotation angles (*θ*) from 0° to 90°

Reference

[1] R. Chakraborty and A. Nag, "Correlation of dielectric confinement and excitonic binding energy in 2D layered hybrid perovskites using temperature dependent photoluminescence," The Journal of Physical Chemistry C, vol. 124, no. 29, pp. 16177-

16185, 2020.
[2] T. Dutta, S. M. Kashid, R. Hooda, T. Sheikh, A. Chowdhury, and A. Nag, "Edge versus Interior Mn2+ Doping in 2D Layered Butylammonium Lead Bromide Perovskite Single Crystals," The Journal of Physical Chemistry C, vol. 126, no. 49, pp. 21109-21116, 2022.
[3] W. Liu et al., "Giant Two-Photon Absorption and Its Saturation in 2D Organic–Inorganic Perovskite," Advanced Optical Materials, vol. 5, no. 7, p. 1601045, 2017.
[4] F. O. Saouma, D. Y. Park, S. H. Kim, M. S. Jeong, and J. I. Jang, "Multiphoton absorption coefficients of organic–inorganic lead halide perovskites CH3NH3PbX3 (X= Cl, Br, I) single crystals," Chemistry of Materials, vol. 29, no. 16, pp. 6876-6882, 2017.
[5] F. Zhao et al., "Comparison Studies of the Linear and Nonlinear Optical Properties of CsPbBr x I3–x Nanocrystals: The Influence of Dimensionality and Composition," The Journal of Physical Chemistry C, vol. 123, no. 14, pp. 9538-9543, 2019.
[6] R. A. Ganeev et al., "Strong nonlinear absorption in perovskite films," Optical Materials Express, vol. 8, no. 6, pp. 1472-1483, 2018.
[7] G. Walters et al., "Two-photon absorption in organometallic bromide perovskites," ACS nano, vol. 9, no. 9, pp. 9340-9346, 2015.
[8] F. O. Saouma, C. C. Stoumpos, M. G. Kanatzidis, Y. S. Kim, and J. I. Jang, "Multiphoton absorption order of CsPbBr3 as determined by wavelength-dependent nonlinear optical spectroscopy," The journal of physical chemistry letters, vol. 8, no. 19, pp. 4912-4917, 2017.
